# Non-coding RNAs regulation of macrophage polarization in cancer

**DOI:** 10.1186/s12943-021-01313-x

**Published:** 2021-02-01

**Authors:** Swati Mohapatra, Carlotta Pioppini, Bulent Ozpolat, George A. Calin

**Affiliations:** 1 Department of Translational Molecular Pathology, The University of Texas MD Anderson Cancer Center, Houston, TX USA; 2 The University of Texas MD Anderson Cancer Center UT Health Graduate School of Biomedical Sciences (GSBS), Houston, TX USA; 3 Department of Experimental Therapeutics, The University of Texas MD Anderson Cancer Center, Houston, TX USA; 4 Center for RNA Interference and Non-coding RNAs, The University of Texas MD Anderson Cancer Center, Houston, TX USA; 5 Life Science Plaza, Suite: LSP9.3012, 2130 W, Holcombe Blvd, Ste. 910, Houston, TX 77030 USA

**Keywords:** Macrophages, MicroRNAs, Long noncoding RNAs, Polarization, Cancer

## Abstract

Noncoding RNA (ncRNA) transcripts that did not code proteins but regulate their functions were extensively studied for the last two decades and the plethora of discoveries have instigated scientists to investigate their dynamic roles in several diseases especially in cancer. However, there is much more to learn about the role of ncRNAs as drivers of malignant cell evolution in relation to macrophage polarization in the tumor microenvironment. At the initial stage of tumor development, macrophages have an important role in directing Go/No-go decisions to the promotion of tumor growth, immunosuppression, and angiogenesis. Tumor-associated macrophages behave differently as they are predominantly induced to be polarized into M2, a pro-tumorigenic type when recruited with the tumor tissue and thereby favoring the tumorigenesis. Polarization of macrophages into M1 or M2 subtypes plays a vital role in regulating tumor progression, metastasis, and clinical outcome, highlighting the importance of studying the factors driving this process. A substantial number of studies have demonstrated that ncRNAs are involved in the macrophage polarization based on their ability to drive M1 or M2 polarization and in this review we have described their functions and categorized them into oncogenes, tumor suppressors, *Juggling* tumor suppressors, and *Juggling* oncogenes.

## Background

The high throughput state-of-art technologies have made the transcriptome more easily accessible, which allowed the discovery of the RNA transcripts that do not encode for proteins termed “non-coding RNAs (ncRNAs)”, which constitute up to 98% of the transcribed human genome [1]. The complex interactions between various types of ncRNAs and other DNA/RNAs, proteins, and lipids [2] can explain their role as regulators in many cellular pathways including immunological processes. Furthermore, they are abnormally regulated in several dreadful diseases including many cancer types [3]. The broad classification of ncRNAs is based on their size and divided into two major groups: small ncRNA (< 200 bp) and long ncRNAs (lncRNAs) (> 200 bp) [4]. The small ncRNAs class includes mainly microRNAs (miRNAs), small nucleolar RNA (snoRNA), small interfering RNA (siRNAs), small nuclear RNA (snRNA), transfer RNAs (tRNAs), and Piwi-interacting RNAs (piRNAs) [5]. While small ncRNAs like miRNAs are highly studied for almost two decades, investigation on lncRNAs realm started in the last decade. Depending on their location in the genome, lncRNAs can be classified into multiple categories, such as natural antisense transcripts (NATs) overlapping in antisense usually protein coding genes, enhancer-like ncRNAs (eRNAs) from the enhancer regions, transcribed ultra-conserved regions (T-UCRs) from the ultra-conserved elements, transcribed pyknons (T-PYKs from human-specific regions) [6] and long intergenic ncRNAs (LINC) from regions between protein-coding genes [7–9]. lncRNAs have important roles as gene regulators in post-transcriptional and transcriptional, epigenetic chromatin remodeling, protein functioning, small RNAs’ maturation, and other significant biological processes including macrophage polarization occurring from monocytes [10, 11]. Monocytes are precursor cells of macrophages produced from progenitor cells in the bone marrow called monoblasts that are produced from hematopoietic stem cells [12]. Monocytes are released into the bloodstream and migrate to the tissues at the periphery, thereby undergoing differentiation into macrophages and dendritic cells depending on their unveiling to the microbial compounds, inflammation-favoring cytokines, and growth factors [10].

In this review, we summarize recent findings regarding ncRNAs (miRNAs and lncRNAs) that mechanistically regulate the differentiation process of macrophages, based on their ability to drive M1 or M2 polarization in different cancers and broadly categorize them into oncogenes, tumor suppressors, *Juggling* tumor suppressors, and *Juggling* oncogenes (Fig. 2).

### Macrophage polarization is an event of plasticity

**Fig. 1:**
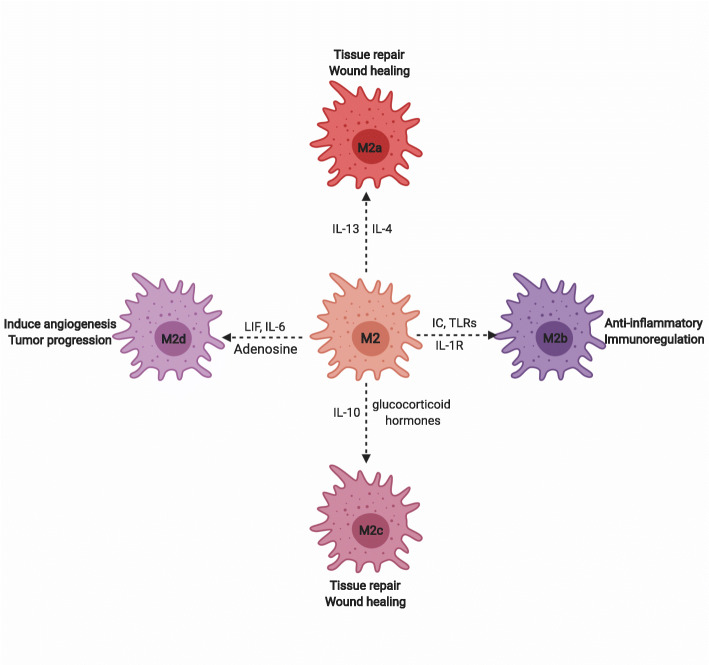
Advanced subtypes of M2 macrophage based on their roles in tumor microenvironment. M2a is known to be induced by IL-13, IL-4 and helps in tissue repair and wound healing. M2b is induced by IC, TLRs, IL-1R and helps in anti-inflammatory effects and immunoregulation. M2c is induced by IL-10 and glucocorticoid and helps in tissue repair and wound healing. M2d is induced by leukemia inhibitory factor (LIF), IL-6, adenosine and helps in the induction of angiogenesis and tumor progression

Macrophages differentiate into specific phenotypes in response to various microenvironmental stimuli and have specific biological functions [11]. Human peripheral blood monocytes (HPBM) are classified on the basis of surface markers CD14 and CD16 into three distinct subgroups: classical monocytes with high CD14 and nil CD16 expression; non-classical monocytes having high CD16 but with comparatively lower CD14 expression; and intermediate type having higher CD14 and lower CD16 expression [13]. Depending on the activation stage and functional status of the macrophages, they have been classified into M1 and M2 types *(*Table 1*)* [14]. In fact, all the three subsets mentioned above if incubated with granulocyte macrophage-colony stimulating factor (GM-CSF) induces M1 type polarization while macrophage colony-stimulating factor (M-CSF) induces M2 type polarization [15]. M1 macrophages are the predominant phenotype in normal immunological responses and involved in TH1 (type I T helper cells) response against different pathogens and produce pro-inflammatory cytokines with tumor-cell and microbe-killing activities [15, 16]. IFN-γ or lipopolysaccharide (LPS) are involved in the classical activation of M1 and in the production of proinflammatory cytokines that leads to phagocytosis of microbes, thereby initiating an immune response while IL-4, IL-13, or IL-10 cytokines help in activating M2 macrophages [17]. M2s induce immunosuppression, tumorigenesis, elimination of parasites, and are involved in wound repair [17]. The oncogene MCT-1 (Multiple Copies in T-cell Malignancy 1) stimulates the secretion of IL-6 that enhances the polarization of THP-1 monocytes into the M2 subtype [18]. The polarization of macrophages can occur at any point during the inflammatory processes and is triggered by various factors including epigenetic, tissue microenvironment; and extrinsic factors like microbial byproducts and inflammatory cytokines [11]. M2 macrophages can be divided into three subgroups namely M2a, M2b, M2c, and M2d (Fig. 1) [19–22]. The M2a subtype differentiation is triggered by mast cells’ secretion of IL-4 and IL-13, Th2 lymphocytes, and basophils [22]. Several mediators such as pro-inflammatory molecules (tumor necrosis factor-alpha (TNF-α), IFN-γ, IL-6, IL-12, IL-1β) and superoxide anions are negatively regulated by IL-4 and IL-13 cytokines, and M2a cells are reported to be involved in tissue repair and wound healing [22]. M2b subtype has anti-inflammatory and immune regulatory roles. In fact, their differentiation is mediated by the interaction between immune complexes (IC) with LPS or IL-1R ligand that reduces the synthesis of IL-12 and increases the production of IL-10 [23]. M2c subtype differentiation is promoted by TGF-β, IL-10, and glucocorticoids. M2c plays an important role in immunosuppression and tissue remodeling. The last subtype, M2d, is activated by the presence of tumor-associated factors. They promote tumor growth and angiogenesis and hence, this subtype is a major constituent of tumor-associated macrophages (TAMs) in the tumor microenvironment (TME) [24]. TME is composed of malignant cells, non-malignant cells, such as immune and stromal cells (i.e, cancer stem cells (CSC), cancer-associated fibroblasts, cancer-associated adipocytes), endothelial cells, fibroblasts, neurons [25, 26], and non-cellular components include extracellular matrix (ECM) proteins, growth factors, cytokines, and metabolites that promotes tumor proliferation, angiogenesis, and metastasis [27–29]. The exact composition of TME differs between different types of cancers. TAMs play an important role in suppressing the immune response and favoring tissue remodeling and consequentially metastasis, and drug resistance [30]. They originate from monocytes after being recruited by factors (i.e, MCP1) released from the stromal and neoplastic cells at the tumor site [16]. Majority of TAMs are associated with Th2 response, secreting IL-10, CCL17, CCL2, CCL22, and TGF-β that favor cancer cell survival, and CCL22 that suppresses antitumor immunity by T_reg_ action [16, 30].

**Table 1:** Important features that help in differentiating M1 from M2 macrophages

Factors	M1 Macrophage	M2 Macrophage
Action	Pro-inflammatory, anti-microbial	Anti-inflammatory, Induce extracellular matrix
Action in tumor-microenvironment	Anti-tumorigenic	Pro-tumorigenic
Activation	Classical (Th1)	Alternative (Th2)
Stimuli	IFN-y, LPS, GM-CSF	IL-4, IL-13, M-CSF, Glucocorticoids
Cytokines and chemokines release	IFN-γ, IL-8, TNF-α, IL-1β, RANTES (CCL5), CXCL10	IL-10, IL-13, CCL17, CCL18, CCL22
Antigen presentation	Yes	No
Nitric oxide and ROS production	High	Low

**Fig. 2:**
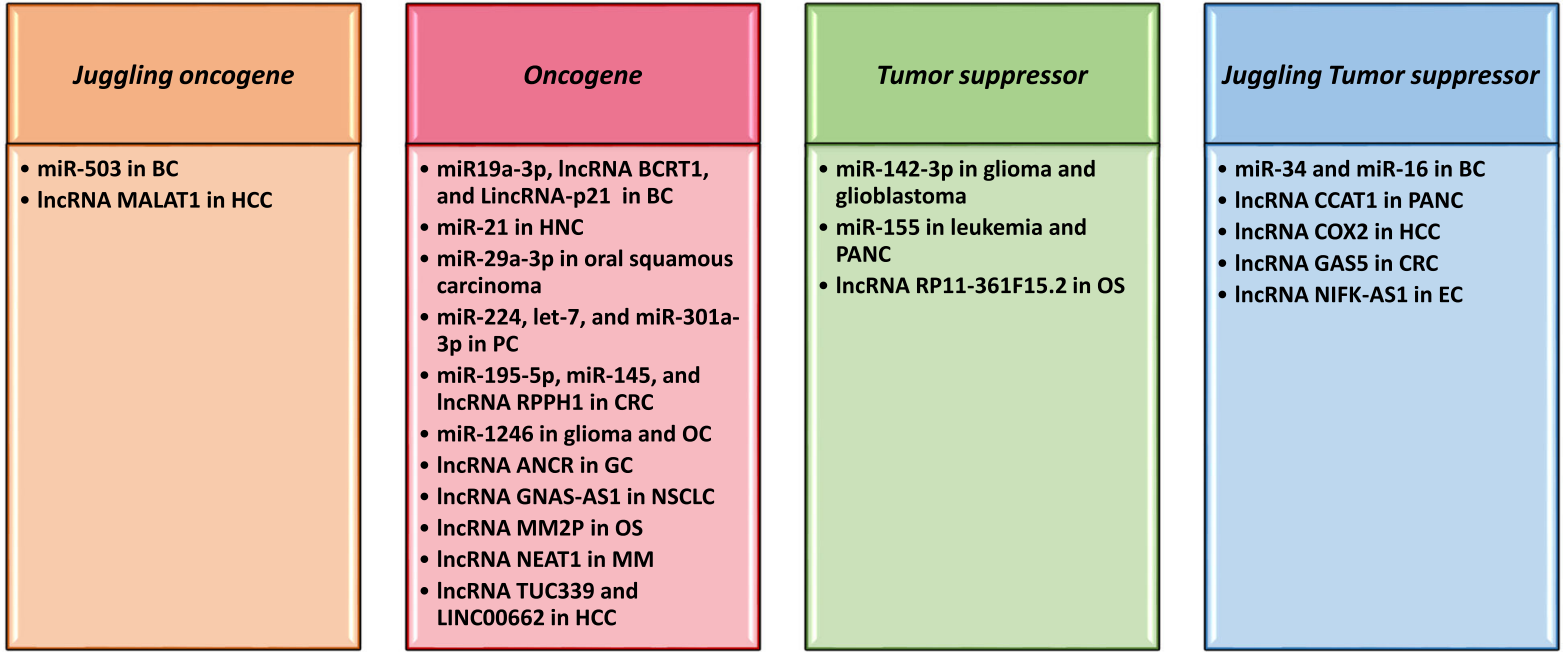
Categorization of ncRNAs into four major types based on their roles in macrophage polarization in different cancer types. This categorization shows the classification of ncRNAs that we have proposed based on their studied roles in polarizing macrophages into M1 or M2 subtype in various types of cancers listed above

From a mechanistic point of view, it has been demonstrated that the equilibrium between activating pathways of STAT1 and STAT3/STAT6 are responsible for the regulation of macrophage polarization and functions [31]. The activation of M1 macrophage polarization is promoted by the increased expression of NF-κB and STAT1, conferring cytotoxic and inflammatory functions. On the other side, the activation of M2 macrophage polarization is mediated by a predominance of STAT3 and STAT6, resulting in immune suppression and tumor progression [32]. Furthermore, peroxisome proliferator-activated receptors (PPAR) such as PPARγ and PPARδ are involved in M2 macrophage activation and oxidative metabolism while KLF4, cooperating with STAT6, induces M2 genes such as *Arg-1*, *Mrc1*, *Fizz1*, *PPAR*γ and inhibits M1 genes (i.e, *TNFa*, *Cox-2*, *CCL5*, *iNOS*) by sequestering NF-κB coactivators. Then, KLF2 majorly regulates macrophage activation by promoting the inhibition of NF-κB/HIF-1α activities [33]. IL-4 leads to induction of c-Myc, thereby providing control over genes such as *Mrc1, Scarb1,* and *Alox15* involved in activating M2 type including other downstream pathways [32]. Also, IL-4 inhibits IRF5-mediated M1 polarization by inducing the M2-polarizing Jmjd3-IRF4 axis. Thereby, IL-10 helps in promoting M2 type polarization by way of inducing the c-Maf, STAT3, and p50 NF-κB homodimer activities [31].

Various reports in cancer have demonstrated a difference in NF-kB activation between early and late stages of cancer progression: a higher NF-kB in M1 macrophage has antitumoral action in initial stages while in the most advanced stages TAMs have a lower NF-kB activation but higher immunosuppression activity [34]. TAM-M2s secrete various pro-angiogenic factors including vascular endothelial growth factor-A (VEGF-A), TNFα and produces different factors responsible for the induction of lymphangiogenesis [35], thereby supporting tumor cell proliferation, angiogenesis, invasion, and cancer stem cell functions [36]. Recent studies have shown ncRNAs to be one of the key regulators of TME, cancer progression, and cancer cell signaling through exosomes [27, 37]. Exosomes are a type of extracellular vesicles that play a significant role in establishing intercellular communication network between cancer cells, tumor immune and stromal cells [38]. Exosomes carrying tumor associated cargo such as ncRNAs are demonstrated to have the potential of becoming novel biomarkers and targets in progression of cancer as they play significant roles in inducing angiogenesis, metastasis, immune response, and therapeutic resistance [38].

### microRNAs regulating M1 or M2 macrophage-type polarization in different cancers

miR-16 expression was reported to be upregulated by epigallocatechin gallate (one of the polyphenols in green tea having anti-tumorigenesis effect) in 4 T1 murine breast cancer (BC) cells and involved in repolarization of M1 macrophages by regulating the TAMs in the TME through reduction of IL-6 and TGF-β and high TNFα [39]. The findings elucidated the anti-tumor activity of miR-16 by inhibiting TAM infiltration and M2 polarization thereby promoting the repolarization of M1-like macrophages [39].

miR-19a-3p is involved in inducing the M2- like macrophage phenotype by regulating the Fra-1 proto-oncogene involved in breast tumor progression and invasion [40]. Overexpression of miR-19a-3p induces inhibition of BC progression and metastasis through macrophage regulation, indicating the potential of therapeutic applications using this miRNA [41]. miR-19a-3p is also involved in the promotion of colitis-associated colorectal cancer (CRC) by regulating the TNFα loops which were majorly found in macrophage and dendritic cells [40]. However, the mechanism behind miR-19a-3p in the induction of M2-like macrophages has not been elucidated to date [40].

miR-21 containing exosomes increased the M2 polarization compared to M1 and were found to be engulfed by CD14+ human monocytes [42]. miR-21 knockdown in the Snail-expressing head and neck cancer (HNC) cells also reduced the Snail-induced M2 polarization which further mitigated the tumor growth and angiogenesis. miR-21 repressed the expression of programmed cell death protein 4 (PDCD4) and IL12A which resulted in M2-type macrophage polarization. The authors provided significant findings that shed light on epithelial to mesenchymal(EMT)-mediated M2 polarization mechanisms potentially serving as a tumor progression biomarker and a good target for the TME remodeling mediated through EMT [42].

miR-29a-3p is often upregulated in the exosomes derived from oral squamous cell carcinoma (OSCC) cells. Exosomes enriched in miR-29a-3p are transported to the unpolarized macrophages and are differentiated into M2-subtype through the activation of SOCS1/STAT6 signaling, eventually leading to invasion and tumor cell proliferation [43].

miR-34a was reported for the inhibition of MCT-1-promoted IL-6R expression and the polarization of M2 which depicts the potential of miR-34a in driving M1 polarization [44, 45]. Another report also demonstrated that the expression of miR-34a in triple-negative BC mediate M1 polarization while antagomiR-34a promotes M2 plasticity [18]. miR-34a is also known for stimulating CRC invasion and metastasis by the IL6R/STAT3/miR-34a feedback loop through its interaction with STAT3 and IL-6R [45].

miR-142-3p is a tumor-suppressor and supports the M1 phenotype of macrophage polarization. This miRNA is often overexpressed in the gliomas and has been demonstrated to be involved in apoptosis of M2 macrophage and inhibits glioma-infiltrating macrophages which in turn inhibits glioma growth [46]. It was reported that miR-142-3p is downregulated in glioblastoma infiltrating macrophages and M2 type showed lower expression of miR-142-3p [46]. M2 macrophages overexpressing miR-142 selectively modulate the transforming growth factor beta receptor 1 that triggered apoptosis of M2 type subset [46].

miR-145 plays an important role in polarizing M2-like macrophages in CRC cells [47]. CRC cells secreting miR-145 in extracellular vesicles were involved in promoting the M2-like phenotype by downregulating the class of enzymes that remove acetyl groups from histone (including histone deacetylase 11) [47]. These have been further demonstrated to be involved in growth of tumors in mice and miR-145 was found to be acting as a communication tool between the TAMs and cancer cells, leading to induction of tumor-promoting TME [47].

miR-155 has been one of the most extensively studied miRNAs in different cancers and is found to be involved in driving the M1 polarization of the macrophages which are generally known to be pro-inflammatory in action [48, 49]. Recently, exosome-mediated expression of miR-155 and miR-125b-2 were reported to be involved in repolarization to M1 macrophages in the transfected pancreatic cancer (PANC) cells [50]. Furthermore, it was shown that modified tumor derived exosomes can reprogramme macrophage polarization in PANC TME [50].

miR-195-5p is involved in tumor suppression in CRC by directly regulating the NOTCH2 expression and thereby inhibiting CRC metastasis and M2-like polarization of macrophages. In turn, miR-195-5p/NOTCH2 was found to inhibit the M2-like TAMs polarization by suppressing the GATA3-mediated IL-4 secretion pathway in CRC cells, in turn helping in M1-type polarization. In summary, miR-195-5p/NOTCH2-GATA3/IL-4 axis can lead to be an impending therapeutic target [51].

miRNA-224 is involved in the inhibition of the progression of prostate cancer (PC) by downregulation of TRIB1 [52]. It was further reported that TRIB1 is responsible for the induction of M2 like-macrophages by IKB-zeta inhibition in the PC that was validated both in vitro and in vivo in mice models [53]. In summary, miR-224 downregulates TRIB1, which induces M2 phenotype macrophages and leads to the inhibition of the IKB-zeta in PC.

miR-301a-3p: The hypoxic exosomal miR-301a-3p promotes metastasis phenotype in PANC cells by inducing differentiation of M2 macrophage from the stromal macrophage [54]. The level of miR-301a-3p was enhanced under hypoxia conditions in both the PC cell-derived exosomes and PANC cells [54]. The genes responsible for the abovementioned regulation were HIF-1a and HIF-2a [54]. Further, knocking down miR-301a-3p in exosomes weakened the polarization of macrophages into M2 type significantly, thereby reducing invasion and the migration capability, and metastatic ability of the PANC cells both in vivo and ex vivo [54].

miR-503 was shown to play a vital role in promoting brain metastasis by programming the microglia inducing M1 to M2 macrophage polarization in BC patients [55]. All these events are initiated when XIST (X-inactive-specific transcript) expression is decreased in BC that activates MSN-c-Met which increases tumor cell stemness [55]. XIST knockout in the mammary glands of mice was significantly promoting the primary tumor growth and metastasis in the brain. These investigations validated the role of miR-503 in regulating M2 polarization in the microglia [55].

miR-1246: Hypoxic glioma cell-derived exosomes dispense miR-1246 in inducing the M2-like macrophage polarization [56], while in ovarian cancer (OC) miR-1246 confers chemoresistance by targeting through the M2-type macrophage polarization [57]. The target of miR-1246 in glioma cancer cells was TERF2IP via the NF-kB and STAT3 pathways [56] while in OC it was mediated through Cav1/p-gp/M2-type macrophage axis [57].

miRNA Let-7b has an important function in the modulation of macrophage polarization, which in turn enhances the presence of TAMs in PC [58]. The upregulation of miR-let-7b was a signature event in PC-TAMs and downregulation in TAMs changed the inflammatory cytokines expression profiles [58]. The magnitude of this function was high, and it led to a reduction of the mobility and angiogenesis of prostate carcinoma cells when treated with let-7b inhibitors [58]. Let-7 expression can be suppressed by STAT3 through the direct instigation of LIN28A/LIN28B expression by the process of inflammation-stimulated EMT [59]. Let-7c regulates inflammation and associated cytokines through sputum macrophages in the initiation and progression of lung cancer (LC) [60]. Recently it was reported that the Lin-28B-let-7-HMGA2 axis was involved in inducing BC stem cells by M1 macrophage activation [61]. miR-let-7a is shown to be involved in M2 macrophage polarization and enhances oxidative phosphorylation activity in the hypoxic melanoma B16-F0 cells which were further engulfed into bone marrow macrophages [62].

### Long non-coding RNAs regulating M1 or M2 macrophage-type polarization in cancers

lncRNA ANCR, an anti-differentiation ncRNA, is significantly upregulated in gastric cancer (GC). The overexpressed lncRNA ANCR inhibited FOXO1 expression and affected macrophage polarization [63]. Previous studies showed that FOXO1 is mediator of the production of inflammatory factors by macrophages favoring M1 macrophage polarization and hence, leading to anti-tumor environments [64]. The upregulation of lncRNA ANCR in GC enhanced the migration and the invasion of the tumor, inhibiting macrophage M1 by targeting FOXO1 and by reducing the concentrations of IL-6 and IL-1β in the cells [63].

lncRNA BCRT1 is upregulated and correlated with poor prognosis through its involvement in tumor growth and metastasis in BC patients. It was shown that lncRNA BCRT1 acts as a tumor promoter by competitively binding to miR-1303 and thereby preventing the degradation of the miRNA target gene PTBP3, a tumor promoter in BC [65]. Overexpression of lncRNA BCRT1 promotes M2 macrophage polarization through exosome-mediated transfer experiments [65].

lncRNA CCAT1(colon cancer-associated transcript-1) has been studied in several cancer types and shown to be overexpressed in GC, multiple myeloma (MM), and BC [66]. lncRNA CCAT1 promotes cell proliferation and migration by interacting with miR-148a and regulates PIK3IP1 in OS [67]. lncRNA CCAT1 is highly expressed by M1 macrophages than TAM (M2-like) macrophages. Furthermore, M1 macrophages are characterized also by a higher expression of PKCζ that is negatively correlated with miR-148a expression level because PKCζ is the target gene of that miRNA [68]. In PC, miR-148a has been reported to be upregulated [69], while the expression of lncRNA CCAT1 has been reported to be varying by IL-4 stimulation. lncRNA CCAT1 downregulation promotes polarization towards to M2 and tumor invasiveness by increasing miR-148a expression that favored M2 macrophage polarization by decreasing PKCζ expression level [68].

lncRNA Cox-2 expression is induced by toll-like receptors (TLR) and it regulates the expression of different genes such as *Ccl5*, *Tlr7*, *Stat1*, *IkB*, and *Icam1* involved in immune regulations [70]. It is also regulated by the nuclear antisense ncRNA PACER, transcribed 300 bp upstream COX-2 transcriptional start site, that interact with the homodimer NF-kB repressor p50/p50, inhibiting its interaction with the promoter and favoring the activating interaction of heterodimer p50/p65 that induce lncRNA Cox-2 transcription [71]. lncRNA Cox-2 has been studied in HCC and is highly expressed in M1 macrophages compared to M2 macrophages [72]. Inhibition of lncRNA Cox-2 decreases levels of certain cytokines including IL-12, iNOS, and TNFα in M1 macrophages and increases the levels of Arg-1, IL-10, and Fizz-1 in M2 macrophages [73, 74]. lncRNA Cox-2 normally inhibits EMT, evasion of immune cells, migratory, and invasive abilityof HCC cells advancing M1 macrophage polarization and inhibiting the M2 macrophage phenotype. Inhibition of Cox-2 can setback the inhibition of M1-type macrophages on tumor proliferation, metastasis, migration, and invasion while increasing the polarization to M2 macrophage in HCC simultaneously [72].

lncRNA GAS5 is involved in the inhibition of tumor progression in different cancer types such as CRC, non-small-cell lung carcinoma (NSCLC), low-grade gliomas, and GC [75–78]. lncRNA GAS5 has a role in macrophage polarization in CRC macrophages and is highly expressed in M1 macrophages. Its inhibition has been shown to promote macrophages towards M2 phenotype, suggesting its role in polarization to M1. Nevertheless, recent reports demonstrated that lncRNA GAS5 inhibits the CRC cell proliferation, promotion of apoptosis and migration [76]. However, the mechanism of action of this lncRNA seems to be ambiguous and needs to be further elucidated [76]. Some studies showed that GAS5 is correlated with poor prognosis in several cancer types including NSCLC and GC [77, 78]. Later studies suggested that lncRNA GAS5 can be used as a predictor of patient survival in glioblastoma brain cancer patients [79, 80]. Low expression of lncRNA GAS5 has also been to be associated with poor survival in low-grade glioma [81].

lncRNA GNAS-AS1 is overexpressed in TAMs, tumor tissues, cell lines and positively correlates with poor patient survival and metastatic free survival in NSCLC patients [82]. lncRNA GNAS-AS1 can promote M2 polarization and enhance expression of TAM markers (IL-10 and Arg-1) [82]. lncRNA GNAS-AS1 induces NSCLC cell proliferation, migration, and invasion through its ability to inhibit miR-4319 and thereby promoting the expression of miR-4319 that targets as N-terminal EF-hand calcium-binding protein 3 (NECAB3) [82]. GNAS-AS1-overexpression induces macrophage differentiation of THP-1-monocytic cells and promotes cell viability, migration, and invasion of NSCLC cells [82]. The role of GNAS-AS1 as a promoter of M2 polarization has been also studied in ER-positive BC [83]. GNAS-AS1 is overexpressed in ER+ breast cancer tissues and M2 macrophages. GNAS-AS1 mediates sponging of miR-433-3p and accelerates M2 macrophage polarization and promotes cell proliferative, migration, and invasive ability of ER-positive breast cancer cells [83]. One of the targets of miR-433-3p is GATA3 that could be positively regulated by GNAS-AS1 [83].

lncRNA LINC00662 is mainly located in the cytoplasm and its expression is similar to the levels of miR-15a/16/107 [84]. LINC00662 acts as an oncogene in different human cancers such as acute myeloid leukemia (AML), GC, PC, LC [84–87], and HCC [88]. This lncRNA has strong expression in HCC tissues when matched to normal liver tissues and its expression is associated with poor prognosis [88]. LINC00662 can interact as ceRNA that binds to miR-15a, miR-16, and miR-107, inhibiting their action on the target genes and inducing the activation of Wnt/β-catenin signaling in HCC cells and macrophages [88]. In HCC cells, activation of Wnt/β-catenin signaling occurs in an autocrine mode and it induces the proliferation and invasion ability in HCC cells while in macrophage the Wnt/β-catenin signaling is activated in paracrine mode, thereby promoting M2 polarization [88].

lncRNA MALAT1 (Metastasis-Associated Lung Adenocarcinoma Transcript 1) plays a vital role in the developmental stages of various cancer types as liver cancer [89] and renal carcinoma [90]. It plays a role as a modulator of angiogenesis and immune response in several cancer types, including human neuroblastoma [91] and thyroid cancer by modulating the secretion of fibroblast growth factor (FGF2) from TAM promoting angiogenesis [92]. In HCC cells, MALAT1 and VEGF-A are both overexpressed compared to healthy normal cells. The interaction between VEGF-A and miR-140 and between miR-140 and MALAT1 is validated where lncRNA MALAT1 acts as a molecular sponge against miR-140 [93]. MALAT1 has a negative correlation with miR-140, hence, the inhibition of miR-140 in HCC induces angiogenesis in HCC cells while favoring macrophage M2 polarization [93].

lncRNA MM2P: According to the lncRNA microarray-based profiling assay, lncRNA-5730422e09Rik, then named lncRNA MM2P, has been shown to promote M2 polarization [94]. During polarization of M2-type macrophages, this lncRNA was upregulated while in contrast was downregulated during the M1-type polarization. Furthermore, knocking down of lncRNA MM2P blocked the cytokine-driven M2-type macrophage polarization and reduced the strength of the angiogenesis-promoting characteristics of M2-type macrophages by inhibiting the STAT6 phosphorylation [94]. lncRNA-MM2P may be able to maintain the activation of STAT6 and so it might be involved in macrophage M2 polarization [94].

lncRNA NEAT1 (nuclear paraspeckle assembly transcript 1) is overexpressed in many cancerous conditions and exerts oncogenic functions [95]. Furthermore, it regulates the accumulation of miR-214 by molecular sponging in endometrial carcinoma cells [96]. lncRNA NEAT1 induces tumor cell proliferation and induces M2 TAM polarization via molecular sponging of miR-214 and regulates the expression of B7-H3 (immune checkpoint regulator) [97]. B7-H3 can also regulate M2 macrophage polarization via the JAK2/STAT3 signaling but the underlying molecular mechanism has not been fully elucidated yet [98].

lncRNA NIFK-AS1 is often downregulated in the TAMs in endometrial cancer (EC) [99]. NIFK-AS1 overexpression inhibits IL4-induced M2 polarization by NIFK-AS1/miR-146a through targeting Notch1 that helps in the expression of pro-inflammatory cytokines in M1 macrophages [99]. Overexpression of miR-146a favors the expression of M2-inducing genes like IL-10 and Arg-1, thereby affecting tumor growth and TAM recruitment [100]. LncRNA NIFK-AS1 negatively regulates miR-146a, increasing the expression of Notch1 in macrophages and so it is involved in estrogen-induced proliferation, invasiveness, and migration of EC cells. However, overexpression of miR-146a reduces the inhibitory action of NIFK-AS1 on M2 polarization suggesting the important role of both these ncRNAs in the M2 polarization process [99].

LincRNA p21 has been studied in several diseases including cancers such as NSCLC where it’s involved in angiogenesis and has been linked to poor prognosis in resected NSCLC patients [101]. It also plays a vital role in atherosclerosis where it is found to modulate p53-dependent target genes [102]. LincRNA p21 plays a critical role in p53/MDM2 stability by directly interacting with p53 and abolishing MDM2 degradation to p53. This event favors the M2-like TAM polarizing phenotype in BC patients [103]. LincRNA p21 knockout reverses the M2-like TAM phenotype towards an anti-tumor function, mediated by the interaction of p53 with MDM2 leading to induction of NF-κB and STAT3 signaling pathway activation [104].

lncRNA RPPH1 is upregulated in CRCs and associated with poor patient prognosis [105]. RPPH1 can promote tumor cell migration and invasiveness in CRC. LncRNA RPPH1 interacts with TUBB3 and inhibits its ubiquitination, leading to enhancement of its stability. TUBB3 activates the Snail pathway and induces EMT that promotes metastasis. RPPH1 is also transported through exosome to macrophages in TME favoring their M2 polarization and its upregulation is observed in the plasma of CRC patients, suggesting its potential as a therapeutic target and diagnostic marker in CRC [105]. lncRNA RPPH1 acts as an oncogene also in BC [106] and acute myeloid leukemia (AML) [107].

lncRNA RP11-361F15.2 is highly expressed in osteosarcoma (OS) tissues and correlated with cytoplasmic polyadenylation element binding protein 4 (CPEB4) [108]. lncRNA RP11-361F15.2 acts as a ceRNA that binds to miR-30c-5p, blocks the inhibitory action of this microRNA on CPEB4 and thereby increases its expression levels [108]. The upregulation of CPEB4 leads to increased cell motility and migration, proliferation, and invasive ability in OS [108]. Upregulation of RP11-361F15.2 and CPEB4 is responsible for increase in the expression of many polarization markers of M2 such as CD206, Arg1, and Ym1 [108]. All together RP11-361F15.2 fosters tumor progression through CPEB4 and inhibits M2-Like polarization of TAMs using miR-30c-5p in OS [108].

lncRNA TUC339 is an HCC-derived exosomal lncRNA [109] involved in regulating the macrophage polarization of M1/M2 in the TME. This lncRNA has a positive association with IL-4 (M2-type) macrophage polarization. Its overexpression is linked to the downregulation of the FcR-mediated phagocytosis pathway and the actin cytoskeleton pathway, thus concedes phagocytosis. TUC339 overexpression also affects cell motility and migration of macrophage’s progenitors, reduces the pro-inflammatory cytokines production while decreasing co-stimulatory molecules expression by the interaction with THP-1 monocytic cells [110].

lncRNA XIST act as an oncogene by promoting tumor growth and EMT in several cancer types such as colorectal cancer and retinoblastoma where it is demonstrated to be a marker of poor prognosis [111–113]. In LCs, it has been demonstrated that lncRNA XIST plays an essential role for invasion, migration and proliferation of LC cells by modulating miR-186-5p [55]. Furthermore, the expression of lncRNA XIST is regulated by TCF-4 (Transcription factor 4) and it positively regulates M2 macrophage polarization making themselves potential targets for gene therapy in LCs [114].

### Complexities and contradictions in this field

ncRNAs are functionally categorized as regulatory molecules that are involved in many cellular processes. They can interact with each other and generate a complex regulatory network that can affect target genes [2, 3, 115]. These properties make the ncRNA biology more complex and harder to understand their differential role in cancer cells and TME, as some ncRNAs act as tumor suppressors or oncogenes depending on the target genes, cancer type and the genetic make-up of the cells [116]. Regarding the roles of ncRNAs in regulating M1/M2 polarization, some of them show dual macrophage polarization behavior in different cancer types (Table 2). Let7a-5p is involved in M1 macrophage reprogramming in lung adenocarcinoma cells [119, 120] while let-7a in hypoxic melanoma B16-F0 cells is involved in M2 macrophage polarization and enhances oxidative phosphorylation activity through regulation of mTOR signaling pathway in macrophages [62, 121]. miR-21 is involved in TLR activation, activation of pro-inflammatory cytokine signaling, and NF-kB in NSCLC cells [122] and CRC cells which are M1-type functional characteristics of macrophages [123]. However, in glioma cells, miR-21 has been reported to induce proliferation, tumor promotion, and immunosuppression that shows M2-type functional characteristics [124].

**Table 2:** Summary of all the ncRNAs that are currently reported to be involved in regulating macrophage polarization in different types of cancer

***ncRNA***	Cancer type	Macrophage polarization	Targets/signaling pathways	Categorization	Reference
***miR-34-a***	BC	Promote M1 Polarization, Inhibit M2 Polarization	IL-6R, STAT3	Juggling Tumor Suppressor	[44, 45]
***miR-16***	BC	Promote M1 Polarization, Inhibit M2 Polarization	IL-6, TGFβ, TNFα	Juggling Tumor Suppressor	[39]
***miR-503***	BC	Promote M2 Polarization, Inhibit M1 Polarization	STAT3 phosphorylation, NF-kB	Juggling Oncogene	[55]
***miR-19a-3p***	BC	Promote M2 Polarization	Fra-1 proto-oncogene, TNFα, NF-kB	Oncogene	[40, 41]
***lncRNA GNAS-AS1***	BC, NSCLC	Promote M2 Polarization	p53	Oncogene	[82, 83]
***LincRNA p21***	BC	Promote M2 Polarization	miR-1303 sponging, PTBP3	Oncogene	[101–104]
***lncRNA BCRT1***	BC	Promote M2 Polarization	miR-433-3p sponging, IL-10 and Arg-1	Oncogene	[65]
***miR-Let-7b***	PC	Promote M2 Polarization	IL-12, IL-23, TNFα	Oncogene	[58–62]
***miR-224***	PC	Promote M2 Polarization	TRIB1, IKB-zeta	Oncogene	[52, 53]
***lncRNA CCAT1***	PC	Promote M1 Polarization, Inhibit M2 Polarization	lncRNA CCAT1/miR-148a/PKCζ axis	Juggling Tumor Suppressor	[66–68]
***miR-301a-3p***	PANC	Promote M2 Polarization	PTEN/PI3Kgamma	Oncogene	[54, 117]
***miR-155***	PANC, leukemia	Promote M1 Polarization	C/EBPβ	Tumor Suppressor	[48–50]
***miR-1246***	glioma, OC	Promote M2 Polarization	NF-kB-STAT3-TERF2IP axis	Oncogene	[56]
***miR-142-3p***	glioma	Promote M1 Polarization	TGF-β	Tumor Suppressor	[46, 118]
***lncRNA LINC00662***	HCC	Promote M2 Polarization	Wnt/β-catenin	Oncogene	[84, 85, 88]
***lncRNA MALAT1***	HCC	Promote M2 Polarization, Inhibit M1 Polarization	miR-140, VEGF-A	Juggling Oncogene	[92, 93]
***lncRNA TUC339***	HCC	Promote M2 Polarization	IL-1 β, TNFα	Oncogene	[109, 110]
***lncRNA COX-2***	HCC	Promote M1 Polarization, Inhibit M2 Polarization	IL-12, iNOS, and TFN-alpha(M1), Arg-1, IL-10, and Fizz-1(M2)	Juggling Tumor Suppressor	[71, 72]
***miR-145***	CRC	Promote M2 Polarization	IL-12p40, IL-10, HDAC11	Oncogene	[47]
***miR-195-5p***	CRC	Promote M2 Polarization	miR-195-5p/NOTCH2-GATA3/IL-4 axis	Oncogene	[51]
***lncRNA GAS5***	CRC	Promote M1 Polarization, Inhibit M2 Polarization	IRF4	Juggling Tumor Suppressor	[75, 76]
***lncRNA RPPH1***	CRC	Promote M2 Polarization	TUBB3	Oncogene	[105–107]
***lncRNA MM2P***	OS	Promote M2 Polarization	STAT6	Oncogene	[94]
***lncRNA RP11-361F15.2***	OS	Inhibit M2 Polarization	miR-30c-5p, CPEB4	Tumor Suppressor	[108]
***miR-29a-3p***	OSCC	Promote M2 Polarization	SOCS1/STAT6 signaling	Oncogene	[43]
***miR-21***	HNC	Promote M2 Polarization	PDCD4, IL12A	Oncogene	[42]
***lncRNA NIFK-AS1N***	EC	Promote M1 Polarization, Inhibit M2 Polarization	NIFK-AS1/miR-146a/NOTCH1 axis	Juggling Tumor Suppressor	[99]
***lncRNA ANCR***	GC	Inhibit M1 Polarization	FOXO1	Oncogene	[63]
***lncRNA NEAT1***	MM	Promote M2 Polarization	lncRNA NEAT1/miR-214/B7-H3/JAK2-STAT3 axis	Oncogene	[95, 96]
***lncRNA XIST***	LC	Promote M2 Polarization	IL-4, TCF-4	Oncogene	[111, 114]

Among the lncRNAs involved in M1/ M2 polarization, some have both tumor-suppressive or oncogenic behavior depending on cancer type; for example, lncRNA ANCR regulates M1 polarization in GC by promoting migration and invasion of tumors. However, some of the previous studies demonstrated that ANCR inhibits tumor metastasis by acting as a tumor suppressor in different cancer types as CRC [125], BC [126], and NSCLC [127]. In NSCLC, ANCR acts as an upstream regulator of TGF-β, the downregulation of which normally promotes EMT, suggesting an oncogenic function [127]. Also, lncRNA NEAT1 induces M2 TAM polarization and promotes proliferation of tumor cells in MM [97], while it exerts tumor suppressive behavior in other cancers such as colon, lung and BC where the reduced expression is associated with poor prognosis by transcriptionally regulating p53-dependent pathways [128]. MALAT1 is one of the most studied lncRNA in the last decade and has been shown to promote M2 polarization via VEGF-A and angiogenesis in HCC [93]. It acts as oncogenes also in many other cancer types, including liver cancer [89] and renal carcinoma [90]. However, in colon and BC, tumor-suppressive role of MALAT1 has been discovered [129]. MALAT1 is a downstream target of PTEN, and it is localized in the nucleus and perinuclear space where it is modulated by PTEN-targeting microRNAs such as miR-17, 20a, and 106b [129]. MALAT1 and PTEN are downregulated in BC and CRC, inhibiting their invasiveness and migration by regulating pro-migratory gene expression, as EPCAM and ITGB4 [129]. All these discoveries emphasize the cancer type-specific role of MALAT1 in tumor development [92]. lncRNA CCAT1 is an essential regulator of M2 polarization and tumor cell invasion in PC, where it’s less expressed while its target gene miR-148a is highly expressed and so it inhibits PKCζ [68]. However, CCAT1 is also well known as an oncogenic lncRNA that is overexpressed in different cancer types as CRC, HCC, OC, LC, GC, and bladder cancer where it enhances tumor cell proliferation, invasiveness, and migration [66]. Studies performed on cancer patients of the abovementioned types also demonstrated that an elevated expression of the lncRNA CCAT1 correlated with poor prognosis [130]. Overall, these studies suggest that the role of ncRNAs in macrophage polarization in different cancers are complex and need to be extensively studied in multiple types of cancer that will help in better understanding and in proposing a new systematic categorization.

### *Juggling* tumor suppressors vs *Juggling* oncogenic ncRNAs

**Fig. 3:**
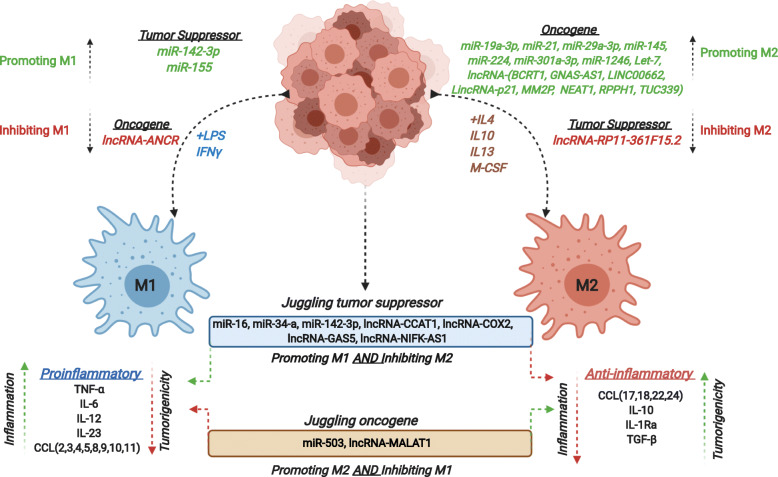
Regulation of macrophagic polarization by ncRNAs (miRNAs and lncRNAs) reported in different types of cancers. The schematic figure illustrates ncRNAs acting as oncogenes or tumor suppressors that play an important role in macrophagic activation and polarization. The cytokines driving the M1 and M2 polarization are listed. ncRNAs acting as tumor suppressors or oncogenes in driving the M1 and M2 polarization respectively have been shown. The “juggling” oncogenes or tumor suppressors have dual roles as mentioned in the figure. The pro-inflammatory and anti-inflammatory cytokines listed are produced by M1 and M2 macrophages respectively after polarization that further regulate the downstream processes. Red: Inhibit; Green: Promote

As described above, various ncRNAs play different roles in TAM-M2 polarization in cancer. We have denoted them as “***Oncogenes***” for the ncRNAs that promote M2 polarization or inhibit M1 polarization. This category includes miR-19a-3p, miR-21, miR-29a-3p, miR-145, miR-195-5p, miR-224, miR-301a-3p, miR-1246, miR-let-7, lncRNA BCRT1, lncRNA GNAS-AS1, LINC00662, LincRNA-p21, lncRNA MM2P, lncRNA NEAT1, lncRNA RPPH1, and lncRNA TUC339 for M2 promotion and lncRNA ANCR for M1 inhibition. We denoted another ncRNA category as “***Tumor Suppressor***” for all those ncRNAs that promote M1 polarization or inhibit M2 polarization such as miR-142-3p and miR-155 for M1 promotion and lncRNA RP11-361F15.2 for M2 inhibition. The other two categories of ncRNAs are designated as “***Juggling Tumor Suppressors***” or “***Juggling Oncogenes***”; the juggling tumor suppressors are ncRNAs that promote M1 and inhibit M2 polarization while the juggling oncogenes promote M2 and inhibit M1 polarization (Fig. 2) because the juggling attribute describes the multitasking abilities of these ncRNAs. The juggling tumor suppressor ncRNA category includes miR-16, miR-34a, miR-142-3p, lncRNA CCAT1, lncRNA COX2, lncRNA GAS5, and lncRNA NIFK-AS1 while the category of juggling oncogene includes miR-503 and lncRNA MALAT1. We should also consider the fact that a juggling oncogene can be a tumor suppressor and vice versa in malignant cells (Fig. 3).

The above classification can be influenced by the increased knowledge of each ncRNA and so further studies about ncRNAs role in macrophage polarization in cancer could lead to more additions to our classification. For example, discoveries could convert a simple tumor suppressor into a juggling tumor suppressor depending on the new findings about the functional role of ncRNAs. However, no reported studies were found yet in the literature that demonstrated the role of other ncRNAs (excluding miRNAs and lncRNAs) in regulating macrophagic polarization in different cancers, although few ones are related to non-cancer diseases [131].

## Conclusion and future directions on ncRNA-therapeutics

In the past decade, the therapeutic applications of ncRNAs not only in cancer but also in other deadly human diseases have significantly increased in numbers and first-in-human miRNA-therapeutics were initiated and tested in phase I clinical trials and recently moved into Phase II clinical trials for advanced tumors, including Cobomarsen (locked nucleic acid based oligo-inhibitor of miR-155) in T-cell Leukemia/ Lymphoma [132], TargomiR (miR-16 mimic based therapy) in mesothelioma [133] and Miravirsen (anti-miR-122) in individuals infected with hepatitis C virus (HCV) [134]. ncRNAs can have the potential to be used as regulators of macrophage polarization and reprogramme TME as well as modulate the immune response as several studies have reported that ncRNAs regulating the gene expression are involved in immune cell function and immune checkpoint inhibitors, thereby mediating the immune responses in various cancers [135]. In fact, currently miRNA mimics and antagonists capable of reprogramming TME are being tested in clinical trials in humans as a novel class of promising therapeutic strategies [136]. The miRNA mimics would act as replacements for downregulated endogenous miRNAs. For example, miR-138 mimic can specifically target and bind to mRNA of PDL1 and CTLA4 and suppress their expressions, mimicking the roles of anti-PD-1 and anti-CTLA-4 antibodies [137].

Manipulation of exosomal lncRNAs to target tumors and TME is another interesting therapeutic strategy owing to the exceptional biocompatibility and biodistribution of extracellular vesicles which can be used for the delivery of therapeutic cargo or modulator ncRNA delivery into tumors [138]. It is also interesting to underline that in addition to miRNAs, lncRNAs can also be thrown into the extracellular space and have potential to get detected in the body fluids. This can be exploited further to develop potential circulating ncRNAs biomarkers that can reflect the expression of secreted body fluids in normal versus malignant cells [37].

Therefore, there is a clear need to broaden the ncRNA horizon which demand more investigations for the proper elucidation of novel ncRNAs that are likely to emerge as important regulators of immunological processes including macrophage polarization and potential molecular targets and therapeutic tools related to their oncogenic or tumor suppressor functions in various human cancers.

## Data Availability

Not applicable

## Abbreviations

BC: Breast cancer
Cav-1: Caveolin-1
CCAT1: Colon cancer-associated transcript-1
COX-2: Cyclooxygenase-2
C/EBPβ: CCAAT/enhancer-binding protein β
CPEB4: Cytoplasmic polyadenylation element binding protein 4
CRC: Colorectal cancer
CSC: Cancer stem cells
EC: Endometrial cancer
ECM: Extra cellular matrix
EMT: Epithelial-mesenchymal transition
eRNAs: Enhancer-like ncRNAs
FGF-2: Fibroblast growth factor-2
FOXO1: Forkhead box protein O1
Fra-1: Fos-related antigen-1
GAS5: Growth arrest specific 5
GATA3: GATA Binding protein 3
GC: Gastric cancer
HCC: Hepatocellular carcinoma
HIF: Hypoxia-inducible factor
HMGA2: High Mobility Group AT-Hook 2
HDAC-11: Histone deacetylase 11
HNC: Head and neck cancer
IC: Immune Complex
IFN: Interferon
IKB: Inhibitor NF-Kappa B
IL: Interleukins
INOS: Inducible nitric oxide synthase
IRF: Interferon regulatory factor
JAK2: Janus Kinase 2 protein
JMJD3: Jumonji domain-containing protein D3
KLF: Kruppel-like factor
LC: Lung cancer
LIF: Leukemia inhibitory factor
LINC: Long intergenic ncRNAs
LNA: Locked nucleic acid
lncRNA: Long noncoding RNA
LPS: Lipopolysaccharide
MALAT1: Metastasis-Associated Lung Adenocarcinoma Transcript 1
MCP1: Monocyte chemoattractant protein-1
M-CSF: Macrophage colony-stimulating factor
MCT-1: Multiple Copies in T-cell Malignancy 1
MDSC: Myeloid-derived suppressor cells
MHC: Major histocompatibility complex
miRNA: MicroRNA
miRNAs: MicroRNAs
MM: Multiple myeloma
MMP: Metalloproteinase
MRC-1: Mannose receptor C-type 1
MSN-c: Met- c-Met via MSN-mediated protein stabilization
NATs: Natural antisense transcripts
ncRNA: Noncoding RNA
NEAT1: Nuclear paraspeckle assembly transcript 1
NECAB3: N-terminal EF-hand calcium- binding protein 3
NF-kB: Nuclear factor kappa-light-chain-enhancer of activated B cells
NOTCH2: Neurogenic locus notch homolog protein 2
NSCLC: Non-small cell lung cancer
OC: Ovarian cancer
OS: Osteosarcoma
OSCC: Oral squamous cell carcinoma
PACER: p50-associated COX-2 extragenic RNA
PANC: Pancreatic cancer
PC: Prostate cancer
PDCD4: Programmed cell death protein 4
PIK3IP1: Phosphoinositide-3-kinase interacting protein 1
piRNA: Piwi-interacting RNA
PKC: Protein kinase C
PPAR: Peroxisome proliferator-activated receptor
PTBP3: Polypyrimidine tract binding protein 3
RPPH1: Ribonuclease P RNA component H1
rRNA: Ribosomal RNA
siRNAs: Small interfering RNA
snoRNA: Small nucleolar RNA
snRNA: Small nuclear RNA
SOCS1: Suppressor of cytokine signaling 1 protein
STAT: Signal transducer and activator of transcription protein
TAMs: Tumor-associated macrophages
TCF-4: T-cell specificTranscription factor 4
TERF2IP: Telomeric repeat-binding factor2-interacting protein 1
TH1: Type I T helper cells
TME: Tumor microenvironment
TGFβ: Tumor growth factor-beta
TNFα: Tumor necrosis factor alpha
T-PYKs: Transcribed pyknons
TRIB1: Tribbles homolog 1
tRNAs: Transfer RNAs
TUBB3: Tubulin Beta 3 Class III protein
T-UCRs: Transcribed ultra-conserved regions
VEGF-A: Vascular endothelial growth factor-A
XIST: X-Inactive Specific Transcript
